# Genetic parameters for milk production and type traits in North American and European Alpine and Saanen dairy goat populations

**DOI:** 10.3168/jdsc.2023-0389

**Published:** 2023-11-04

**Authors:** Marc Teissier, Luiz F. Brito, Flavio S. Schenkel, Guido Bruni, Pancrazio Fresi, Beat Bapst, Christèle Robert-Granie, Hélène Larroque

**Affiliations:** 1 GenPhySE, Université de Toulouse, INRAE, ENVT, F-31326, Castanet-Tolosan, France; 2 Department of Animal Sciences, Purdue University, West Lafayette, IN 47907; 3 Centre for Genetic Improvement of Livestock, Department of Animal Biosciences, University of Guelph, Guelph, ON, Canada, N1G-2W1; 4 ARAL, Crema, Italy 26013; 5 AssoNaPa, Roma, Italy 00187; 6 Qualitas AG, Zug, Switzerland 6300

## Abstract

The development of an across-country genomic evaluation scheme is a promising alternative for enlarging reference populations and successfully implementing genomic selection in small ruminant populations. However, the feasibility of such evaluations depends on the genetic similarity among the populations, and therefore, high connectedness and high genetic correlations between the traits recorded in different countries or populations are needed. In this study, we evaluated the feasibility of performing an across-country genomic evaluation for milk production and type traits in Alpine and Saanen goats from Canada, France, Italy, and Switzerland. Variance components and genetic parameters, including genetic correlations between traits recorded in different countries, were calculated using combined phenotypes, genotypes, and pedigree datasets. The (co)variance component analyses were performed within breed, either based only on pedigree information or also incorporating genomic information. Across-country genetic parameters were calculated for 3 representative traits (i.e., milk yield, fat content, and rear udder attachment). The heritability estimates ranged from 0.10 to 0.50, which are consistent with previous estimates reported in the literature. The genetic correlations for rear udder attachment ranged from 0.75 (between France and Italy, for the Alpine breed without genomic information) to 0.95 (between Canada and France, for the Saanen breed with genomic information), whereas for fat content, between France and Italy, they ranged from 0.75 in the Alpine breed without genomic information to 0.78 in the Alpine breed with genomic information. However, genetic correlations for milk yield were only estimable between France and Italy, with a moderate value of 0.45 for the Alpine breed with or without genomic information, and of 0.22 and 0.26 in the Saanen breed with and without genomic information, respectively. These low genetic correlations for milk yield could be due to several factors, including the trait definition in each country and genotype-by-environment interactions (GxE). The high genetic correlations found for fat content and rear udder attachment indicate that these traits might be more standardized across countries and less affected by GxE effects. Thus, an international genomic evaluation for these traits might be feasible. Further studies should be performed to understand the surprisingly lower genetic correlations between milk yield across countries. Furthermore, additional efforts should be made to increase the genetic connection among the Alpine and Saanen goat populations in the 4 countries included in the analyses.

## Highlights

• International genomic evaluations for Canadian, Italian, Swiss, and French Saanen and Alpine dairy goats are feasible for milk production traits and some conformation traits.
• The genetic correlations between countries were high for rear udder attachment (0.75 to 0.92) and fat content (0.75 to 0.78).
• The moderated genetic correlations for milk yield between France and Italy need to be further investigated due to the importance of this trait in the breeding objectives of both populations.

## Graphical Abstract

**Figure:**
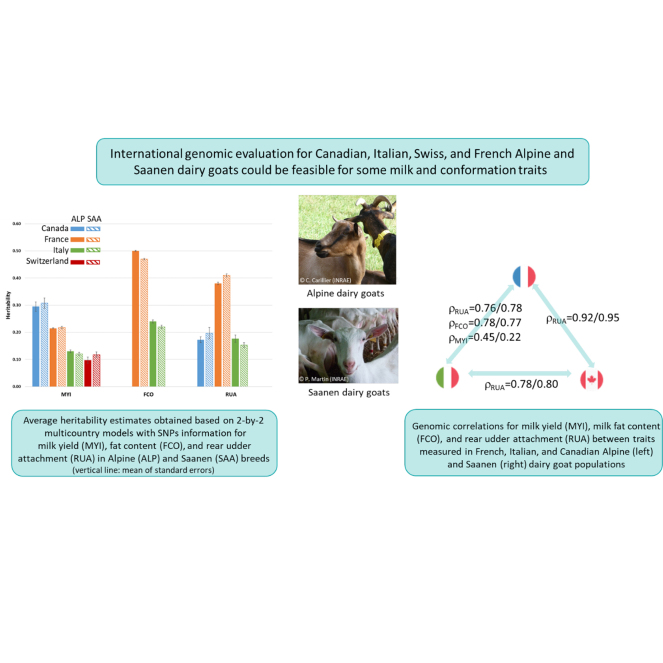
**Summary:** A potential strategy for implementing genomic selection in small populations consists of sharing genomic and phenotypic datasets for developing across-country genomic prediction of breeding values. The quality of these genomic evaluations depends on the genetic relationship among the populations from the countries involved. In this context, this study evaluated the feasibility of an international genomic evaluation for Alpine and Saanen goat populations from 4 countries involved in the European SMARTER project, including results of genetic parameters across countries. The strong genetic correlations estimated between countries for rear udder attachment and fat content are encouraging for future international genomic evaluations.

Over the past decades, international genetic evaluations have been developed for both dairy and beef cattle populations (Bonifazi et al., 2022; Nilforooshan and Jorjani, 2022). This practice has contributed to an intensification in the rate of exchange of genetic material, as importing countries have an evaluation of the expected performance of the bulls' progeny in their population and environment (Nilforooshan and Jorjani, 2022). In the context of genomic selection, across-country evaluations enable the enlargement of reference populations, and thus, more reliable genomic breeding values can be obtained (Brøndum et al., 2011). In addition, breeding values can be compared across countries.

Across-country evaluations are still incipient in small ruminants, although, on a more modest scale than in cattle, there is also exchange of genetic material between countries. Recently, Fitzmaurice et al. (2022) reported strong genetic correlations for body weight and carcass composition traits in Texel sheep from Ireland and the United Kingdom. The authors also reported an increase in the rate of genetic gain using a selection of sires independently of the country of origin. Furthermore, de Oliveira et al. (2022) evaluated the feasibility of performing across-country genomic predictions in Norwegian White Sheep and New Zealand Composite sheep populations with similar development history, and the authors concluded that across-country genomic predictions might be possible for those populations, especially for traits recorded only in New Zealand or Norway. Although these studies were done in sheep and none exist in goats, single country genomic prediction of breeding values have also been performed in goat populations (Carillier et al., 2013; Rupp et al., 2016; Teissier et al., 2019; Massender et al., 2022a,b). However, the size of the reference populations is often small and combining datasets from different countries could increase the prediction reliability of genomic breeding values, especially for lowly heritable or difficult-to-measure traits. Therefore, France, Italy, Switzerland, and Canada, which are part of the H2020 SMARTER Project (www.smarterproject.eu), shared phenotypes, genotypes, and pedigree datasets for both Alpine and Saanen dairy goat breeds to evaluate the feasibility of an across-country genomic evaluation for these 2 goat breeds. A first study has confirmed the exchange of genetic material between these countries, which were mainly limited in exports of genetic material from France to the other 3 countries (Teissier et al., 2022). The genomic analyses have shown that European dairy goat populations, especially French and Italian, are more closely related at the genomic level, whereas Canadian dairy goat populations are more genetically distant from the European goat populations (Teissier et al., 2022). Interestingly, the Canadian dairy goat populations seem to be more admixed (Brito et al., 2015, 2017). The main objective of this study was to estimate (co)variance components for 2 milk production traits (i.e., lactation milk yield [**MYI**] and average milk fat content [**FCO**]), and an udder type trait (i.e., rear udder attachment [**RUA**]), in the Alpine and Saanen goat breeds from France, Canada, Italy, and Switzerland. These selected traits represent the main breeding goals in the countries involved and different levels of trait heritability.

The 4 countries shared MYI; Canada, France, and Italy shared RUA; and Italy and France shared FCO records. This study was exempt from formal institutional animal care and use approval because data were recorded in each country according to normal practices (agronomic or veterinary practice) in commercial farms. Table 1 presents a description of the phenotypic records shared by each country. Raw phenotypes (instead of de-regressed breeding values) were shared to enable the use of common statistical methods for calculating breeding values based on datasets from different countries. Milk production records of goats born between 2000 and 2010 for France and between 2002 and 2015 for Italy were retained. Phenotypes recorded from 1978 to 2018 and from 1988 to 2019 were available for Canada and Switzerland, respectively. In addition, only data from goats whose sires had at least 6 offspring were selected. The RUA was recorded in Canada, France, and Italy based on a scale from 1 to 9 (Manfredi et al., 2001). The mean of this score, for the 2 breeds (weighted by the number of phenotypes available for each breed), was close between Canada (5.60) and Italy (5.75) and a little weaker in France (4.63) for both breeds. The FCO is expressed in grams per kilogram of milk in France and in decigrams per kilogram of milk in Italy. In France the observed FCO phenotypic mean was 37.05 g/kg for Alpine and 35.08 g/kg in Saanen against 339.78 dg/kg for Alpine and 325.45 dg/kg in Saanen in Italy. In contrast, the mean of MYI in kilograms was different across the 4 countries. The total amount of milk in France was calculated for a reference lactation length of 250 d with a mean of 872.53 kg in Alpine and 874.66 kg in Saanen. In Canada, a 305-d lactation was fitted based on a random regression test-day model, with an average MYI of 949.47 kg for Alpine and 1,118.93 kg in Saanen. Switzerland derived 2 milk traits because some of the goats were dried off after 100 DIM, before the availability of the alpine summer pasture: from 1 to 100 DIM and from 101 to 200 DIM. Therefore, we retained lactations of Swiss animals with milk production during the 2 parts of the lactation and summed up the milk production in the 2 periods. Italy and France adjust the milk yield for the expected lactation duration and extrapolate incomplete lactations. However, Italy calculates milk yield for a reference lactation length of 210 d, and uses a multiplicative pre-adjustment for parity, age, and number of kids. Italy and Switzerland have lower lactation MYI levels than Canada and France (Table 1).

**Table 1:** Summary of data used for the analyses; for each trait within each country and breed: number of pedigree, genotypes and phenotypes retained, and mean, SD, and CV of the phenotypes^1^

Country	Breed	Trait	Pedigree, N	Genotypes, N	Phenotypes, N	Mean	SD	CV
FRA	ALP	FCO	806,877	2,968	1,878,616	37.05	5.17	0.14
ITA	ALP	FCO	63,617	1,061	150,162	339.78	54.31	0.16
CAN	ALP	MYI	8,007	793	7,414	949.47	284.43	0.30
CHE	ALP	MYI	25,497	1,280	66,794	565.74	222.62	0.39
FRA	ALP	MYI	807,534	2,968	1,882,056	872.53	262.72	0.30
ITA	ALP	MYI	71,276	1,061	170,932	524.02	203.95	0.39
CAN	ALP	RUA	9,434	793	5,235	5.49	1.43	0.26
FRA	ALP	RUA	474,963	2,968	326,423	4.50	1.50	0.33
ITA	ALP	RUA	29,721	1,061	22,757	5.59	1.56	0.28
FRA	SAA	FCO	665,202	2,009	1,463,568	35.08	5.02	0.14
ITA	SAA	FCO	76,741	338	161,920	325.45	54.13	0.17
CAN	SAA	MYI	4,238	903	3,947	1,118.93	427.15	0.38
CHE	SAA	MYI	25,857	503	64,376	614.51	242.26	0.39
FRA	SAA	MYI	665,538	2,009	1,464,961	874.66	277.03	0.32
ITA	SAA	MYI	85,633	338	189,395	549.15	215.61	0.39
CAN	SAA	RUA	4,796	903	2,443	5.82	1.41	0.24
FRA	SAA	RUA	303,709	2,009	203,668	4.84	1.67	0.34
ITA	SAA	RUA	34,288	338	26,548	5.90	1.71	0.29

1 ALP = Alpine breed; SAA = Saanen breed; CAN = Canada; FRA = France; ITA = Italy; CHE = Switzerland; FCO = average milk fat content (g/kg in France; dg/kg in Italy); MYI = lactation milk yield (kg); RUA = rear udder attachment (scores from 1 to 9).

The size of the pedigree files shared by each country was proportional to the number of phenotypes, with France having the largest number of recorded individuals, followed by Italy, Switzerland, and Canada (Table 1). The maximum pedigree depth was 31, 31, 35, and 23 generations for France, Canada, Switzerland, and Italy, respectively. The average pedigree depth was 4.01 in Canada, 3.74 in France, 2.19 in Italy, and 8.45 in Switzerland. The pedigree completeness also differed across countries, with 4%, 8%, 22%, and 35% animals with missing parents in Switzerland, Canada, France, and Italy, respectively. The average (and maximum) number of offspring per sire was 25 (3,443), 13 (256), 11 (191), and 8 (80) in France, Italy, Switzerland, and Canada, respectively. Only French and Swiss animals were found in several pedigree files. France exported genetic material to all countries, but did not import animals from any of them. In contrast, Italy and Canada did not export animals to any other countries. The proportion of animals with French sires was 7.20%, 1.54%, and 0.58% in Italian, Swiss, and Canadian dairy goat pedigrees, respectively (Teissier et al., 2022).

The 4 countries also shared data from animals genotyped using the Goat SNP50 BeadChip (Illumina Inc., San Diego, CA). A quality control was performed on these genotypes using the PLINK 1.9 software (Purcell et al., 2007), which consisted of excluding SNPs with a call rate lower than 0.90 or a minor allele frequency lower than 0.01, and animals with call rate lower than 0.90. The quality control was done within breed and country and the data were subsequently merged. A total of 9,855 animals and 50,578 SNPs were kept for further analyses. The genotypes of French animals represented about 50% of the total amount of genotypes in both breeds. Swiss genotyped animals comprised 21% and 8% of the Alpine and Saanen animals, respectively. These proportions were of similar magnitude for Italian animals (18% of Alpine and 8% of Saanen), but were 13% and 25% for Canadian Alpine and Saanen animals, respectively.

Variance-covariance components were computed via Gibbs sampling, using the gibbs1f90 software (Misztal et al., 2002). The analyses were run using either only phenotypic and pedigree information or also incorporating genomic information. A multitrait animal model was fitted for each trait and breed, where each country was considered as a different trait. The systematic effects included in the statistical models resembled the ones used in routine genetic evaluations in each country. For milk production traits, the models included the animal genetic and permanent environment as random effects. The fixed effects for France included an interaction of herd, year, and parity; an interaction effect of age at kidding, parity, year, and region; an interaction effect of month of kidding, parity, year, and region; and an interaction effect of the length of the dry period, parity, year, and region. The fixed effects for Italy included the interaction effect of herd, year, and season of kidding. For Switzerland, the fitted fixed effects were parity and an interaction effect of year and season, whereas herd was considered as a random effect. The fixed effects for Canada included herd, parity, and year. For RUA, the models contained the random animal genetic effect. The fixed effects included in the models for analyzing the French datasets were the interaction effect of herd, year, and parity; the interaction effect of age at scoring-parity and year; and the interaction effect of lactation stage at the scoring-parity by year. The fixed effects fitted for the Italian datasets included an interaction between DIM and parity, classifier, herd-season of classification, and age as linear and quadratic covariates. The models for the Canadian datasets also included an interaction of herd, year, and classifier as a random effect, and parity, age, and DIM as fixed effects.

Figure 1 shows the average heritability estimate for each trait obtained in bivariate analyses when including genomic information. We obtained several heritability estimates for a specific country as we ran all 2-by-2 country combinations and the estimates were similar regardless of the analyses. The heritability estimates had an average standard error (SE) of 0.01 with a minimum of 0.00 and a maximum of 0.04. An average difference of 0.05 points was observed between heritability estimates from pedigree and genomic analyses. For France, the heritability estimates for the Alpine and Saanen breeds were close to the estimates routinely used in the genetic evaluations, except for MYI, with a slightly lower heritability of about 0.22 compared with 0.30 in the French national evaluations (Larroque et al., 2011; Carillier et al., 2014). For Italy, the estimates of heritability for RUA are of the same order of magnitude as those usually used in the routine genetic evaluations (0.18 vs. 0.16 in the Alpine evaluations and 0.15 vs. 0.10 in the Saanen evaluations; Biffani et al., 2020). The heritability estimates for FCO in Italian goats were 0.24 and 0.22 in Alpine and Saanen breeds respectively, whereas a heritability of 0.44 is routinely used in Alpine and Saanen genetic evaluations. Similarly, the heritabilities estimated for MYI were 0.13 in Alpine and 0.12 in Saanen, whereas values of 0.25 and 0.24, in Alpine and Saanen breeds respectively, are currently used in the routine genetic evaluations. For Canada, the heritability estimates calculated are consistent with those used in their routine genetic evaluations for MYI (~0.30 for both breeds; Massender et al., 2022a). However, the average estimates obtained for RUA (0.17 for Alpine and 0.20 for Saanen) are lower than the estimates used in the routine genetic evaluation (0.45 for both breeds). In Switzerland, MYI had a heritability of 0.10 and 0.12 for the Alpine and Saanen breeds, respectively, which are similar to the estimates used in their routine genetic evaluation (~0.15; Bapst et al., 2013). Overall, the MYI heritability estimates ranged from 0.10 (Swiss Alpine) to 0.31 (Canadian Saanen) for both breeds. A higher heritability estimate of 0.56 has been reported for MYI in the Yorkshire dairy goat population (a composite breed originally consisting of Alpine, Saanen, and Toggenburg; Mucha et al., 2015). The FCO had a high heritability estimate in French animals (~0.49), but a moderate one in Italian animals (~0.23). The RUA showed comparable heritability estimates between Canada (~0.19 on average for both breeds) and Italy (~0.17 on average for both breeds) and twice as high in France (~0.40 on average for both breeds).

**Figure 1:**
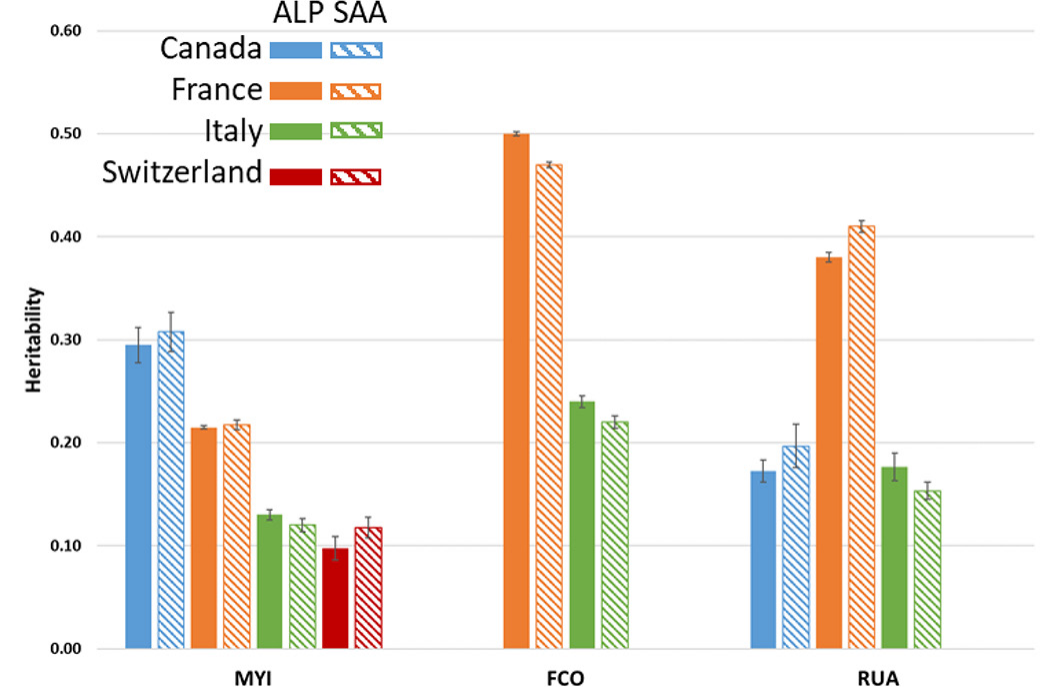
Average heritability of estimates obtained in 2-by-2 multicountry genomic models for milk yield (MYI), fat content (FCO), and rear udder attachment (RUA) in Alpine (ALP, solid bar) and Saanen (SAA, hatched bar) breeds (vertical lines indicate the mean of the SE).

Estimates of (co)variance component and correlations between countries in the Alpine and Saanen breeds using only pedigree information or also adding genomic information are presented in Table 2. For MYI, only the estimates between France and Italy are presented due to the low accuracy (high SE or no convergence of the models) for the correlation estimates with the other countries, likely due to the reduced genetic links across populations (Teissier et al., 2022). Genetic correlations lower than 1 indicate possible genotype-by-environment (**GxE**) interactions, but could also originate from artifacts of the data, such as different data recording and standardization protocols. High genetic correlations (generally greater than 0.70) between countries are a prerequisite to ensure reliable and comparable genetic values across countries (Mulder and Bijma, 2006). The variance estimates were precise and consistent across different preliminary multiple country evaluations performed (i.e., 2-by-2, 3-by-3, or 4-by-4 countries) with or without genomic information. However, the covariance estimates were not consistent across analyses with data from 2, 3, or 4 countries, and had large SE, likely due to the limited links across populations.

**Table 2:** Variances (on the diagonal), covariances (above diagonal), and correlations (below diagonal in bold) for milk yield (MYI), milk fat content (FCO), and rear udder attachment (RUA) in Alpine and Saanen breeds estimated with pedigree only (left number) and with pedigree and genomic information (right number)^1^

Breed	Trait	Country	CAN	FRA	ITA
Alpine	MYI	FRA	NA^2^	9,142/9,180	2,063/2,098
		ITA	NA	**0.45/0.45**	2,320/2,389
	FCO	FRA	NA	9.22/9.21	46.98/49.91
		ITA	NA	**0.75/0.78**	425.78/450.15
	RUA	CAN	0.27/0.25	0.40/0.39	0.21/0.20
		FRA	**0.91/0.92**	0.72/0.72	0.32/0.33
		ITA	**0.81/0.78**	**0.75/0.76**	0.25/0.26
Saanen	MYI	FRA	NA	10,661/10,707	1,322/1,166
		ITA	NA	**0.26/0.22**	2,387/2,416
	FCO	FRA	NA	8.40/8.39	44.68/45.42
		ITA	NA	**0.76/0.77**	406.68/411.03
	RUA	CAN	0.36/0.33	0.56/0.53	0.28/0.26
		FRA	**0.95/0.95**	0.96/0.95	0.43/0.43
		ITA	**0.90/0.80**	**0.84/0.78**	0.27/0.32

1 CAN = Canada; FRA = France; ITA = Italy.

2 NA = not available.

For RUA, the genetic correlations in Alpine ranged from 0.75 between the French and Italian populations without genomic information to 0.92 between French and Canadian populations with genomic information. In the Saanen breed these estimates ranged from 0.78 to 0.95 between the same countries and with genomic information. For all these estimates the SE ranged from 0.00 to 0.05 except between Canada and Italy, in the Saanen breed, in which a SE of 0.43 was observed. For FCO in the Alpine breed the correlation between France and Italy was 0.75 (±0.02) with pedigree information and 0.78 (±0.01) when adding genomic information. In the Saanen breed this correlation was 0.76 (±0.03) with pedigree information and 0.77 (±0.01) with genomic information. For MYI, the correlation between Italy and France was moderate, 0.45 (±0.10) in Alpine with or without genomic information (Table 2). In Saanen, the genetic correlations observed were 0.22 (±0.04) and 0.26 (±0.09) with and without genomic information, respectively. Recently, Garcia-Baccino et al. (2022) found a much higher correlation of 0.70 for MYI between populations of French (Manech) and Spanish (Latxa) dairy sheep, whether from the Blond strain (more genetically close), or from the Black strain (more genetically distinct). The authors presumed that there might be a GxE interaction across these populations and regions.

For RUA, the very similar definition of phenotypes as well as the similar statistical models used in each country are probably the main reasons for the strong genetic correlations between countries. However, the high SE for the correlation between Canadian and Italian Saanen suggests that the small amount of data for these countries combined with a low level of genetic connection between them make it difficult to obtain accurate correlation estimates. We observed lower genetic correlation for FCO than for RUA between Italy and France. Dairy phenotypes (quantity and quality of milk) are collected based on the same protocols with sampling intervals of 4 or 5 wk along the lactations and for all parities. However, phenotypes from Italy are precorrected and the fixed effects included in the genetic models are also different, which could explain these differences in the genetic correlations.

It was surprising that the covariances for MYI across countries were not properly estimated, except between France and Italy. This might be due to the weak genetic connection between dairy goat populations from the 4 countries, except between France and Italy; unbalanced amount of data across datasets (France contributed 88% and 85% of the data for the Alpine and Saanen breeds, respectively); and different data collection protocols across the countries with different heritability estimates and routinely genetic evaluation models.

In summary, the strong genetic correlations estimated between countries for RUA and FCO are encouraging for future international genomic evaluations in dairy goats, especially between France and Italy. However, as milk production is an important part of the selection objectives in dairy goats, it will be necessary to further investigate alternatives for the standardization of the phenotypes to be considered in international genomic evaluations. Furthermore, additional efforts should be made to increase the genetic connection among the Alpine and Saanen goat populations in the 4 countries included in the analyses.
